# Mental health and resilience in young people on Saint Helena Island

**DOI:** 10.1177/13591045241284326

**Published:** 2024-10-15

**Authors:** Tara L Murphy, Elena Nixon

**Affiliations:** 1 Institute of Mental Health, 6123 University of Nottingham, Nottingham, UK

**Keywords:** Saint Helena Island, young people, mental health, resilience, remote geographical context, psychological strengths, british overseas territory

## Abstract

This study explored the mental health needs and strengths of young people (aged 11–18 years) living on the remote island of Saint Helena (SH). 24 young people and their carers completed standardised inventories assessing mental health and resilience, of whom 15 of the young people and their carers participated in semi-structured interviews enquiring into mental health awareness, contributing factors to young people’s mental health and resilience, as well as experience with local mental health services. Descriptive data were reported based on the quantitative measures and thematic analysis was applied to the interview transcripts. A number of young people were found to meet criteria for mental health problems while they showed high levels of resilience. The themes derived from young people’s interviews were broadly centred around mental health boosters, including personal successes, social interactions, engagement in pleasurable activities and use of anxiety management strategies; and around limitations of living in SH in terms of limited resources and difficulty in maintaining trust in the community. From the carers, the themes revolved around awareness of mental health needs and strengths in young people, perceived barriers to access support mainly due to apprehensions around stigma and anonymity. Recommendations for improvement of mental health provision are made.

## Plain Language Summary

Limited research has been carried out on the mental health of young people who live in remote geographical places. This study explored mental health and resilience in a group of young people living on Saint Helena Island. A total of 24 young people and their carers completed questionnaires assessing mental health and resilience and within the group 15 young people and their carers completed interviews with a researcher. We used the scores on the questionnaires to describe the characteristics of the young people and we analysed the interviews for themes denoting common patterns in attitudes, perceptions and behaviours. Young people and their carers reported mental health problems and needs but, positively, high levels of resilience too in young people. The themes derived from young people’s interviews were broadly centred around mental health boosters, including personal successes, social interactions, engaging in pleasurable activities and using anxiety management strategies; and around limitations of living in Saint Helena Island in terms of limited resources and difficulty in maintaining trust in the community. From the side of the carers, the generated themes revolved around awareness of mental health needs and strengths in young people through their observed behaviours, perceived barriers to accessing mental health support mainly due to concerns around stigma and maintaining anonymity, and recommendations for improvement of mental health service provision involving further training and psychoeducation. Implications for future research are discussed such as why the number of people who consented to participate was small and how the findings from the current study could be used to inform and improve child and adolescent mental health service provision on Saint Helena Island.

## Introduction

Epidemiological data on mental health needs in remote populations of overseas territories of the United Kingdom (UK), such as Saint Helena (SH) and the Falklands Islands (FIs) are poorly characterised (Rimicans & McInerny, 2018). A review of mental health data in six Caribbean small island developing states also showed no population-level data on mental health burden with limited capacity to implement mental health policy due to lack of funds and/or staff shortages. In these economically robust islands, child and adolescent mental health services (CAMHS) were rare (Walker et al., 2022). In other remote and rural locations, such as Greenland (Nordic Centre for Welfare and Social Issues, 2017), Canada (Nova Scotia Health, 2017), and Australia (Wainer & Chesters, 2000), the prevalence of mental health disorders are reported to be higher than in urban contexts in the same country, which may be a result of poorer service provision and/or other factors.

SH island is a small and remote UK overseas territory situated in the South Atlantic Ocean. It is an island of limited socio-economic growth, with dependence on the UK for government budgetary aid (Harmer, et al., 2015). The population is 4439 with an average age of 46.9 years that includes 388 young people aged 10–19 years representing 8.7% of the population (2021 census). Wages are low, in 2022/2023, half of all full-time employees earned less than £9, 970 per annum on SH (Saint Helena Government Report, 2024, dated April 2024). The current population from SH have descended from people originating from Asian, African and European nations. People from SH have a strong cultural identity and refer to themselves as ‘Saints’. Reliable and affordable access to the internet has not been available to most of the population on SH until recently (2023). Past screening studies carried out in the 1990s suggest that SH had a population of children with low rates of mental health problems. The purpose of that research was to evaluate the impact of access to television on children’s behaviour and emotional health (Charlton 1997; Charlton et al. 1994, 2000). Of the data collected from teachers of 402 young people aged 9 to 12 years, lower rates (6.9%) of behavioural problems were reported than those reported in comparable cohorts in England (Charlton and Leo, 1994). However, there have been no published studies on mental health in young people on SH since, and importantly, no research to date has captured the voice of young people themselves or that of their families. Similarly, no research has informed the understanding of resilience in this population, thus making the current investigation crucial in terms of shaping up services which meet the needs of young people, inform policy and care.

Healthcare on SH is offered at Jamestown General Hospital with community clinics. Care is provided by nursing staff from the island and overseas, alongside medical, and other health professionals. The expatriate health professionals tend to have fixed term contracts (typically 2–3 years) and when they leave, their specific expertise leaves with them resulting in poorly sustained healthcare. Attempts have been made to transfer learning and skills to local health professional staff, often initially in an apprenticeship role, which are slowly growing in number on the island. In addition, funding and decision-making around funding expenditure varies on the island, which results in poorly sustained healthcare. A CAMHS was recently developed within the community mental health team (CMHT) which is largely delivered by a team of experienced community psychiatric nurses. The CAMHS development was due to an increased recognition of need in children and young people on SH, which was identified as part of the Wass report (2015) and broader objectives regarding children’s welfare within SH (Saint Helena Island Government Report 10-Year Plan, 2017). The CAMHS has had transitional staffing during the past decade and is currently comprised of a CAMHS nurse and a school nurse (on island), and liaises with psychologists and a child psychiatrist through remote consultation from several countries (e.g. England).

### Aims

The overarching aim of the study was to explore the mental health needs and strengths of young people on SH from both perspectives of the young people and their carers/parents, informed by a socio-ecological perspective (Ungar, 2011) and using descriptive and qualitative data. Specifically, the research questions that this study intended to address were: (a) What are the mental health problems and needs of young people? (b) How are psychological strengths manifested in young people? and (c) How are young people’s mental health needs supported by the community and mental health services?

## Methods

### Study design

This study was designed as an exploratory study due to this being an, as yet, unexplored area of research, using standardised quantitative measures to assess mental health needs and resilience, as well as semi-structured interviews to gain further insight into concerns surrounding mental health from the perspectives of young people and their parents/carers. Preparatory Patient and Public Involvement (PPI) was used to plan the study and review the materials. Four young people (11–17 years), two carers and two professionals contributed. A semi-structured interview guide was developed with members of the local mental health team, using a socio-ecological model (Ungar, 2011) drawing on the relevant literature, and items included in the Child and Youth Resilience Measure (CYRM-17; Jefferies et al., 2019).

### Participants

All young people aged 11–18 years (approximately *n* = 350) and their carers were invited to participate. This included young people attending the only secondary school, the community college and those working. Young people identified as having moderate to severe intellectual disability were excluded as they would not have been able to complete the questionnaires.

#### Descriptive characteristics

24 young people and 24 carers were recruited to the quantitative study. The mean age of young people was 14.8 years (11 boys and 13 girls). The carers included 1 grandmother, 2 fathers and 21 mothers.

Fifteen of the young people assented to be interviewed (5 girls and 8 boys aged 11–17 years, mean age: 14.2 years). Fifteen parents/carers participated (2 fathers and 13 mothers; aged 39–55 years, with a mean age = 46.5 years). Purposefully limited information is provided about the participants to maintain the anonymity of those recruited from within the small population of SH.

### Recruitment

Participant information sheets were given by teachers to all school-attending adolescents and their carers to invite them to participate in the research. The study was advertised on local media and presentations at school. Interested young people and/or their carer participants made contact with the researcher via email or telephone to be involved in the study. Assent (young people), consent (carers) and questionnaire completion was carried out within the grounds of the school, the mental health team base or the young people’s home. A subgroup of the sample elected to participate in the face-to-face interviews, which were also carried out at school, the hospital or the young person’s home. Most young people were interviewed alone, aside from two, who elected to be interviewed with their carers. The interviews were carried out immediately following questionnaire completion for 12/15 of the young people, and the remaining 3 interviews were completed within 2 weeks. Young people were offered the option for their participant number to be entered into a draw for a £50 token for a local shop.

For the qualitative component, 15 young people and their carers (*n* = 15) were recruited. This number was deemed to be sufficient for thematic analysis based on previous protocols (Braun & Clarke, 2006) and recruitment was intended to stop when data would reach saturation or when no more participants would offer to take part. All of the data were collected by one researcher (TM). Recruitment was carried out between June 2020 and January 2021. Research governance, approval, and support for the study was gained from the Saint Helena Research Institute and Directors in Saint Helena Government. A standard operating procedure around safeguarding and risk procedures was agreed with the mental health team lead, approved by the University of Nottingham Faculty of FMHS DPAP Research Ethics Committee (Ethics Ref: 2019-1471) and followed throughout the study.

### Measures

Demographic information was collected on age, gender, year at school and relationship between the young person and carer. This tool was selected as it is a well-validated measure that maps on to DSM-5 diagnostic criteria and assesses for the key psychological disorders seen in childhood'. i.e. remove the The MINI-KID 7.0.2 (Sheehan et al., 2010).

The Mini-International Neuropsychiatric Interview for Children and Adolescents (MINI-KID 7.0.2; Sheehan et al., 2010) was completed with young people and their carers in dyads. This tool was selected as it is a well-validated measure that maps on to DSM-5 diagnostic criteria and assesses for the key psychological disorders seen in childhood. It assesses for past and lifetime diagnoses.

#### The Child and Youth Resilience Measure

The Child and Youth Resilience Measure CYRM – 17 (Parent/carer and Child versions; Jefferies et al., 2019) was also administered. The 17-item questionnaire is a standardised measure that assesses resilience and is completed by the child and carer. The simple language version was selected. A three-point scale was used with a score range of 17–51. The tool offers a ‘total’ resilience scale with 2 subscales: *Personal* and *caregiver* resilience. The *Personal* subscale includes 10 items (e.g. “Getting an education is important to me”) whereas the *Carer* subscale includes 7 items (e.g. “I feel safe when I am with my family/caregiver(s)”. There are no cut-off scores published - higher scores are considered to indicate higher resilience.

#### Semi-structured interview

Two interview guides were developed for the study (refer to interview guides). The Young person guide was formulated around questions into awareness/self-care of emotional function/mental health, resilience and coping strategies. The carer/parent guide was formulated around questions related to mental health in young people, resilience and access to CAMHS. The guides followed an open-to-close questioning approach, with initial questions being open, followed by prompts and closed questions, where appropriate, to obtain more information. The interviews lasted for 25–48 minutes for young people and 40–65 minutes for carers. Breaks were used as and when needed.

#### Data analyses

Descriptive statistics were used to summarise the sample characteristics using Excel software. The interviews were transcribed into a Word document and then entered into NVIVO 12, a software analysis package, to facilitate qualitative data coding. Thematic analysis was employed inductively using the six-phase procedure advised by (Braun & Clarke, 2006, 2019). A wide range of codes were applied in the initial analyses, informed by meaning within the content. The two authors co-coded the interviews and agreed the consolidation of the codes to form the categories around which themes and subthemes were formulated.

## Results

Results from the MINI-KID showed that 11 of 24 young people met criteria for one or more psychiatric condition(s) as shown in Table 1. The most commonly reported conditions were tic disorder and anxiety disorders.

**Table 1.** Number of Participants Fulfilling Criteria for a Psychiatric Disorder Based on the MINI-KID.

Psychiatric disorder	Number of participants
Major depression	2
Panic disorder	3
Agoraphobia	2
Separation anxiety	1
Specific phobia	1
Generalised anxiety disorder	2
Tic disorder	4
Attention deficit hyperactivity disorder	1
Oppositional defiance disorder	1
Adjustment disorder	2
Psychotic disorder; social anxiety; mania; suicidality; obsessive compulsive disorder; post traumatic stress disorder; alcohol/substance use disorder; conduct disorder; eating disorder	0

All participants reported high levels of resilience, with similar scores obtained from young people and carers on both personal (child) and carer domains on the CYRM-R (refer to Table 2).

**Table 2.** Participants’ Characteristics Based on Personal and Caregiver Scores on the CYRM–R.

CYRM – R	Mean (SD) range
Child version	
Personal subdomain	2.6 (0.3)
Caregiver subdomain	2.7 (0.21)
Total	46.6 (2.2) 34–51
Carer version	
Personal subdomain	2.7 (0.26)
Caregiver subdomain	2.8 (0.13)
Total	47.7 (1.9) 43–51

### Qualitative findings

Thematic analysis resulted in six themes overall, 3 for young people (with 13 subordinate themes) and 4 for parents/carers (with 10 subordinate themes).

## Young people interviews

The themes (and subthemes) that were derived from the interviews with the young people could be consolidated under two predominant categories: Factors serving as Mental health ‘boosters’; Limitations of living in St Helena (Table 3).

**Table 3.** Themes and Subthemes Derived From Interviews With the Young People.

Categories	Themes	Subthemes
Mental health ‘boosters’	Theme 1: Overcoming daily challenges encourages personal growth	Subtheme 1.1: Success with academic, social and practical tasks acts as a self-booster
		Subtheme 1.2: Self-organisation is seen as personal achievement positively impacting mood
	Theme 2: Social interactions help foster a positive outlook to life	Subtheme 2.1: Relationships with ‘heroes to look up to’ such as parents and tutors positively contribute to mental health
		Subtheme 2.2: Friendships with peers provide a two-way means of psychological support
	Theme 3: Engaging in pleasurable exercise and leisure activities can positively change mood	
	Theme 4: Adopting anxiety management strategies to improve mood	Subtheme 4.1 Improving mood through self-help apps or formal guidance from the internet, such as mindfulness and cognitive behavioural therapy techniques
		Subtheme 4.2 Accessing games and music through the internet helps to feel better
Limitations of living on St Helena	Theme 5: Limited resources restrict exposure to experience	Subtheme 5.1: Lack of financial resources limits engagement with practical activities within the family
		Subtheme 5.2: Lack of availability of resources within the community poses restrictions to what can be done, making life feel monotonous
	Theme 6: Maintaining trust in the community is difficult because ‘everyone knows everyone else’s business’	

### Category: Mental health ‘boosters’

#### Theme 1: Overcoming daily challenges encourage personal growth

##### Subtheme 1.1: Success with academic, social and practical tasks acts as a self-booster

The range of experiences for success was varied and the learning from this success seemed to demonstrate persistence, even in the context of short-term failure.

“I say like, my garden - a simple example. Say like pests or something eat my crops that’s all hard work for nothing, so that make me that just annoy me and then you constantly try to think how can you avoid that next time? In my mind things don’t go your way, so like you working so hard for something but people just throw obstacles at you that can be frustrating and annoying.” (Participant 6).

“I think maybe school exams because they’ve been quite a bit stressful and erm tests themselves, it’s so much information to retain and it can be a struggle and so sometimes you just have to take a step back and just take a breather you know what I mean and then focus on it again” (Participant 12).

##### Subtheme 1.2: Self-organisation is seen as personal achievement positively impacting mood

Being organised helped with stress management and mood and keeping in control of situations and behaviour.

“Umm here like I’m a little bit fussy about umm my room .... it doesn’t have to be super clean but things you know have to be in its place; …and I don’t like feeling crowded, umm sometimes I get really anxious about.” (Participant 2).

#### Theme 2: Social interactions help foster a positive outlook to life

##### Subtheme 2.1: Relationships with ‘heroes to look up to’ such as parents and tutors positively contribute to mental health

The young people could identify several traits and qualities in their parents and teachers that inspired them and they seemed to value and accept advice given directly.

“Umm well yeah, my parents obviously big role models in my life. …. basically just anyone who gives me good advice about my well-being and mentor umm yeah those are my role models actually.” (Participant 3).

“I think if I have a good relationship, I do distance learning courses I’m so.., its nice to connect with the tutors because they not there physically but still connecting with them like just talking about your day or what you did on the weekend is nice before we start the lesson.” (Participant 5).

##### Subtheme 2.2: Friendships with peers provide a two-way means of psychological support

A combination of spending time with other preferred people, combined with a structured activity or enjoying sporting collaboration, helped young people feel good.

“I hang around with a lot of modest people, they are calm, collective, they know how to treat themselves properly and stuff...” (Participant 1).

“Umm my opinion I think a good day is usually spent with family or friends and like not just sitting around, actually doing something like I don’t know like going swimming or something..” (Participant 12).

### Theme 3: Engaging in pleasurable exercise and leisure activities can positively change mood

Leisure and sports activities impacted on young people’s mood dependent on their preferences and developmental stage.

“When I went to the dentist and spend time with mummy. So, like us went ‘Tasty Bites’ to eat, us was talking, us went on the internet together and all that. So like that is fun - when us go for walks and just talk between us.” (Participant 2).

They reported self-regulation of affect and the capacity to enjoy themselves, sometimes in the company of other people, and other times alone.

“Just to go to the library, sit myself read and do whatever work I want or exercise, go for a walk, maybe just listen to music or watch a good movie maybe just something simple or even TV programmes.” (Participant 6).

#### Theme 4: Adopting anxiety management strategies to improve mood

##### Subtheme 4.1: Improving mood through self-help apps or formal guidance from the internet, such as mindfulness and cognitive behavioural therapy techniques

Several young people described using formal anxiety management strategies that they had learnt within school or in school-based support. Supportive strategies to manage mood, including distraction, were also reported, and often these were accessed through the internet and technology.

“We used to get like coping strategies we was given in classes and identifying the stressor and then find a way to you know in the minute, so it was a coping method or even when I counting to 10 sometimes.” (Participant 7).

“Apps - just maybe what I find on the Play store maybe I was putting affirmations, yoga for mornings, relaxing or daily planners or anything - what I find usually its recommended for you.” (Participant 6).

“Well normally, normally at lunch I go on the internet, make me feel a bit better.” (Participant 3).

##### Subtheme 4.2: Accessing games and music through the internet helps to feel better

“Play games, I don’t really ring a lot of people, I text my friends sometimes, but I usually use my phone for playing games” (Participant 8).

“Put a pair of headphones on and listen to music, run around outside and try to get my anger out” (Participant 1)

### Category: Limitations of living on St Helena

#### Theme 5: Limited resources restrict exposure to experience

##### Subtheme 5.1: Lack of financial resources limits engagement with practical activities within the family

The impact of financial constraints of their families and communities was a source of stress and sadness. Financial difficulties were reported to be a source of stress for young people.

“I think its umm mainly finances cos you know erm me and my friends we like to go and do activities but every now and again someone will drop out because their parents don’t have enough money to pay for them...” (Participant 3).

“Their main worry I think is money and expenses of food and just well-being in general” (Participant 6)

##### Subtheme 5.2: Lack of availability of resources within the community poses restrictions to what can be done, making life feel monotonous

Restricted access to entertainment opportunities and clubs available has led to a feeling of monotony and a sense of missing cohesive activities that could serve to bring young people together

“Yeah its not that much to do here, you can’t go to the cinema, you can’t go bowling anything like that, its kind of the same thing over and over again.” (Participant 1).

### Theme 6: Maintaining trust in the community is difficult because ‘everyone knows everyone else’s business’

In the small community, there is a great deal of familiarity between people which according to the young people could result in a lack of trust, hindering them from opening up to other people.

“I talk to people, but trust is hard to find here obviously you got your parents there but other than that I think trust is hard to find.” (Participant 8).

## Parent/carer interviews

The themes (and subthemes) that emerged from the interviews with the parents/carers are centred around three conceptual categories: Awareness of mental health needs and strengths in young people; Perceived barriers to accessing mental health support; Recommendations for improvement of mental health service provision (Table 4).

**Table 4.** Themes and Subthemes Generated From Interviews With Carers.

Categories	Themes	Subthemes
Awareness of mental health needs and strengths in young people	Theme 1: Parents are able to detect mental health needs and strengths in young people	Subtheme 1.1: Observed social cues in young people help provide an explicit manifestation of their emotional and behavioural problems
		Subtheme 1.2: Young people’s temperamental traits and constructive experience are perceived as contributing factors to their adaptability and resilience
Perceived barriers to accessing mental health support	Theme 2: Accessing mental health support remains challenging	Subtheme 2.1: A perceived risk to anonymity hinders help seeking
		Subtheme 2.2: Fear of stigma prevails within a small community despite a positive shift in mental health attitude
Recommendations for improvement of mental health service provision	Theme 3: There is a need to train Saints	Subtheme 3.1: Local nurses trained by the mental health team can play a fundamental role
		Subtheme 3.2: Specialist expertise within mental health teams is needed
	Theme 4: Further psychoeducation about mental health is needed	Subtheme 4.1: Actively reaching out to young people about mental health within community avenues, such as the library
		Subtheme 4.2: Increasing accessibility to mental health in schools through creative ways

### Category: Awareness of mental health needs and strengths in young people

#### Theme 1: Parents are able to detect mental health needs and strengths in young people

##### Subtheme 1.1: Observed social cues in young people help provide an explicit manifestation of their emotional and behavioural problems

Family members reported an awareness of the emotional state of the young people by noticing their internalising and externalising behaviours.

“Er, his behaviour might change, you know he might be secluded, always being in his room or acting out in a different way because they not feeling safe or secure. Not being part of the family. That would be a big sign.” (Participant, 23).

##### Subtheme 1.2: Young people’s temperamental traits and constructive experience are perceived as contributing factors to their adaptability and resilience

Parents highlighted diverse resilient traits in young people and noted how they would learn from their mistakes.

“Er the adaptability because you don’t necessarily have all the things that they got in the UK erm they work quite hard and quite creatively with what they’ve got erm...” (Participant, 30)

“Even his ability to like more or less stand up for himself, his self-esteem and work with problems.” (Participant, 19).

“Yeah he’s just like he always was the child who umm kind of blew us away, if I can say that about him. He never failed to amaze us with knowledge, umm with decisions made in life..” (Participant, 18).

### Category: Perceived barriers to accessing mental health support

#### Theme 2: Accessing mental health support remains challenging

##### Subtheme 2.1: A perceived risk to anonymity hinders help seeking

A key barrier reported by the majority of parents/carers was a perceived problem with keeping personal information private or anonymous, as confidentiality was thought to be difficult to maintain in a small community.

“..umm the goldfish bowl. Your problems are not your own, umm, I don’t know, I shared a bit more with other people. You can’t keep anything secret here.” (Participant 19).

“Well, if we don’t necessarily have mental health help lines whatever, to go into chat rooms with people and talk about their issues knowing that they don’t have to see them again, because here you know, you know everybody in the community” (Participant 25).

##### Subtheme 2.2: Fear of stigma prevails within a small community despite a positive shift in mental health attitude

Stigma around mental health remained a powerful barrier, despite reported attempts to change the attitudes. The perception of people and their judgement were perceived to be a barrier to accessing care.

“I think it’s really important umm to be able to go and speak without being judged. There is still a lot of stigma here about having mental health. Schools try really hard, it doesn’t always work but it’s going to be a slow road to try and help people. To change attitudes of people is the most important thing.“(Participant 18).

### Category: Recommendations for improvement of mental health service provision

#### Theme 3: There is a need to train Saints

##### Subtheme 3.1: Local nurses trained by the mental health team can play a fundamental role

Trained local mental health professionals was thought to be a protective factor in supporting services and mitigating the current problems with the regular change-over in expatriate staffing.

“Shouldn’t we be looking at our local people to try and up skill them well, not necessarily up to the same professional standard as yourself but knowing the basics so that when the new person comes in...” (Participant 23).

##### Subtheme 3.2: Specialist expertise within mental health teams is needed

A differentiation between roles in the mental health team was important.

“….we never had someone like a specialist in that field so you know I think mental health only came the last ten years maybe so all those little mental health issues people used to have, it was just brushed off” (Participant 38).

#### Theme 4: Further psychoeducation about mental health is needed

##### Subtheme 4.1: Actively reaching out to young people about mental health within community avenues, such as the library

The delivery of psychoeducation about mental health could become creative and delivered in community settings to increase accessibility.

“I really think it’s important to reach our youth …and I’m just wondering if we have a little corner somewhere in our clinics or in the library where our young people can say well, I see you’re struggling with something, you know....“. (Participant 31).

##### Subtheme 4.2: Increasing accessibility to mental health in schools through creative ways

Although participants acknowledged existing attempts within schools aimed at increasing mental health awareness, they thought that more creative ways could be adopted to make the education more acceptable.

“Umm even puppet shows going into the schools and it make, adults love it and you know the kids would love it, yeah. You don’t even have to have the mental health team doing it” (Participant 15).

## Discussion

This study investigated mental health and resilience in young people on the remote island of SH. A significant proportion of young people were found to meet diagnostic criteria for at least one mental health disorder. Young people and carers reported strong resilience, emphasising the role of important relationships shared within the family and/or the community alongside intrapersonal and interpersonal factors. These findings were further explored through the themes and subthemes derived from the interviews.

### Mental health problems and needs

A considerable number of young people in the small sample reported symptoms of anxiety, mood disorder and tic disorders. This shows that young people are willing to discuss their needs and were forthcoming in doing so, which may have been part of their motivation for participation. This underlines the importance of including young people in research on their own mental health; as well as an evident need for addressing young people’s mental health needs given the prevalence of mental health problems in this population.

Although the community’s awareness of the importance of mental health has been improving, stigma and lack of trust seems to still impact access to available services and help-seeking behaviour within local society. Kaushik and colleagues (2016) reporting on studies from Australia, Europe and the United States of American also showed that stigma is significant, universal and multifaceted in young people with mental health problems. Themes from carer interviews indicated creative strategies suggested to make available information about mental health by embedding it within the community, in playful ways, presumably to normalise it.

### Resilience

Carers and young people identified psychological strengths such as adaptability, use of strategies/services to manage anxiety, robust and secure relationships and the experience of success which facilitated positive mental health. A systematic review of young people’s mental health self-care showed that the broader literature to date has limited consistency in defining universal self-care (Truscott, 2023) although young people who reviewed the studies highlighted that managing stress/meeting mental health needs and developing life satisfaction were strongly relevant. Insights about the threats to mental health were also identified, including financial constraints and limited resources. Financial struggles for families are reported in studies as a risk factor for poor psychosocial outcome in young people living in non-geographically remote contexts (Devenish et al., 2017); and this theme impacted young people directly in their own lives and also that of their peers, who were unable to access activities that they may have wanted to. Different perspectives were taken on how economic constraints might impact young people’s mental health, whether this was via personal or parental stress or as a direct impact on provision of services.

### Community awareness and support

Besides the overlap in the themes derived from the interviews with the young people and their carers, unique insights were also offered. Young people reported engagement with social support from family and friends was a source of resilience. They highlighted how academic performance needed to be managed in order for it to be a mental health booster while they also recognised the benefits of living in an environment which was safe and facilitative of leisure and exercise. Carers indicated a need for further support through mental health service care on SH and recommendations were made about how to optimise existing care including training. Carers offered creative innovations such as performances which embedded mental health in an accessible way and made relevant literature available in everyday settings, such as the library. The benefits of health professionals who had an ongoing and integrated role in services were recognised. Other remote nations, such as Fiji, have optimised CAMHS expertise through collaborative training between local professionals and external specialists (Robertson et al., 2020). These efforts have focused on cultural dimensions specific to Fiji, considering socio-ecological factors which could be replicated in SH based in part on insights from carers about how this might be suited to Saint Helenian society. In addition, specialist expertise was valued within a diverse team of professionals, which marked the development of services. Hoeft and colleagues (2018) have highlighted how education, supervision and partnership with local communities around mental health could be beneficial in rural and low resource settings which is in line with data from this study.

### Strengths, limitations and future direction

This exploratory study is the first to evaluate mental health and resilience in young people on SH quantitatively and qualitatively. It was possible to recruit participants to engage with an interview-based screening measure, self-report questionnaires as well as semi-structured interviews in a population unfamiliar with research.

Limitations of the study include acknowledgement that the rates of mental health disorder reported may not be representative of the whole population of the island and may be subject to biases subject to the use of the specific measures used. The low uptake in participation suggests that mental health research may be concerning or not of interest to the SH population which resulted in the study being significantly under-powered to carry out statistical analyses on the quantitative data. This may relate to a fear of stigma, which was highlighted as a barrier to mental health care in the findings of the interviews.

Future studies could consider an alternative methodology such as a school and/or workplace screening study in order to capture a fuller understanding of the needs and strengths of a larger popular of young people living on SH. It may also be helpful to understand the impact that greater access to internet connectivity will bring to young people’s perceptions of mental health and resilience, access to remote care and knowledge. In developing this, it will be salient to continue to include the views of young people on mental health so that policy and services can be tailored to meet their needs. A significant emphasis was placed on delivering psychoeducation about mental health to the broader community. Several other important learning points were highlighted to inform this such as drawing on the strengths given from within the community and families themselves, which directly support young people, consideration of the changing role of stigma within the population and appetite for ongoing care from a specialist team of professionals within a society which understands and supports mental health and builds resilience.
